# Reply to May et al. The Goal of Achieving High-Quality Surgical First-Line Therapy in Patients with Penile Cancer Is Important; However, Some Collective Efforts Are Still Required in Order to Reach It. Comment on “Brassetti et al. Combined Reporting of Surgical Quality and Cancer Control after Surgical Treatment for Penile Tumors with Inguinal Lymph Node Dissection: The *Tetrafecta* Achievement. *Curr. Oncol.* 2023, *30*, 1882–1892”

**DOI:** 10.3390/curroncol30040333

**Published:** 2023-04-21

**Authors:** Giuseppe Chiacchio, Aldo Brassetti, Giuseppe Simone

**Affiliations:** 1 Department of Urology, IRCCS “Regina Elena” National Cancer Institute, 00144 Rome, Italy; gipeppo1@gmail.com (G.C.); puldet@gmail.com (G.S.); 2 Department of Urology, Azienda Ospedaliero-Universitaria Ospedali Riuniti di Ancona, Università Politecnica delle Marche, 60126 Ancona, Italy

We have carefully read the comment by May et al. [1] on our novel Tetrafecta, which aims to standardize outcome reporting after partial or radical penectomy with inguinal lymph node dissection (ILND) for penile cancer (PC). The authors noted that our composite outcome may not be suitable for a large portion of PC patients who undergo sentinel node biopsy (SNB). While it is true that the Tetrafecta was not initially designed to describe results after this type of surgery, it could still be used in all pN+ cases detected by SNB that require ILND.

The authors also questioned the arbitrary process that led us to select the four outcomes to define good surgical quality. In contrast, the group of 13 expert PC urologists used a modified Delphi process to derive Pentafecta, which includes adequate indication for organ-preserving surgery (while always aiming for complete tumor excision), proper surgical management of inguinal nodes (depending on cancer stage), timely ILND (when needed), multidisciplinary evaluation of patients to share possible indications for adjuvant chemotherapy, and centralization of PC treatment in referral centers.

While the five selected criteria are aligned with international guidelines and represent the best practices for treating PC patients, there is currently no evidence suggesting that simultaneously meeting these goals provides any clinical advantage for the treated individuals. On the other hand, our study found that achieving the Tetrafecta was associated with improved overall survival outcomes at both 2-year (21% vs. 42%) and 5-year follow-up (24% vs. 43%); the difference was statistically significant (*p* = 0.01). Similarly, achieving the Trifecta after partial nephrectomy [2,3,4] or radical cystectomy with ileal neobladder [5] has also been linked to improved oncologic outcomes.

While involving experts in a Delphi process may be a good way to provide recommendations in the absence of strong evidence, this method was not deemed necessary even when the widely accepted composite outcomes for radical prostatectomy [6], partial nephrectomy [7], and radical cystectomy [8] were developed.

We firmly believe that oncologic surgery should aim for a durable and complete removal of the tumor, while ensuring a smooth post-operative recovery. These principles are indisputable and should be agreed upon by all. Based on these, we selected four standardized and reproducible outcomes that were combined in our Tetrafecta. Achieving negative surgical margins (NSMs) is a cornerstone of partial/radical penectomy, and May et al. also emphasized that PC surgery should always strive for a complete removal of the primary tumor. However, this cannot be histologically proven after certain organ-preserving treatments such as tumor CO2/Nd:YAG laser ablation.

Another criterion shared between our Tetrafecta and the proposed Pentafecta is the significance of adequate lymphadenectomy. While the 13 experts enumerated all the available strategies for treating inguinal nodes according to PC stage (including SNB), we chose to focus on patients who were offered a modified (for staging purposes) or radical (with therapeutic intent) ILND by the urologist. We identified a reliable and reproducible criterion based on cadaveric studies to define the adequacy of the lymphadenectomy: a minimum yield of seven LNs from each treated groin [9] was proposed, since superficial packets contain 4–25 glans (with an average of eight in each extremity), and deeper nodes are not always found [10]. Additionally, we believe that an uneventful postoperative course is essential to define the success of a surgical procedure, particularly in PC surgery where up to one-third of patients experience adverse events that may require reoperation [11] and affect their quality of life. Ultimately, oncologic surgery should aim to produce long-lasting results. In our opinion, the absence of recurrence within 12 months is the minimum requirement to consider surgical treatment satisfactory. Other combined outcomes designed to report results of renal and bladder cancer surgeries also incorporated similar parameters [3,4,8].

In conclusion, despite the methodological limitations that characterized its development, we believe that our Tetrafecta represents a valuable tool for standardizing the reporting of surgical outcomes following partial/radical penectomy with inguinal lymph node dissection. Although it is important to acknowledge that this composite outcome is only applicable to this specific surgical population, achieving the Tetrafecta has been shown to be associated with favorable oncological outcomes, thus highlighting its potential significance.
